# Supplementary material for: Spatiotemporal mapping of rice acreage and productivity growth in Bangladesh
Source: PLoS One. 2024 Mar 15;19(3):e0300648. doi: 10.1371/journal.pone.0300648 (PMC10942088; doi:10.1371/journal.pone.0300648)
Supplement: Attachment — Submitted filename: Response to Reviewer 2.docx [file pone.0300648.s003.docx]

Manuscript number: PONE-D-23-09867

Title: Spatiotemporal mapping of rice acreage and productivity growth in Bangladesh

Journal: PLOS ONE

Dear Reviewer,

Thank you for the comments concerning our manuscript. We deeply appreciate your positive evaluation of our work. Those comments are valuable and very helpful. We have read through comments carefully and have made corrections. Please see below; all tasks and revisions taken are shown point-by-point.

**Response to Reviewer’s comments**

**Reviewer #2:**

**Comment #1:** The research topic “Spatiotemporal mapping of rice acreage and productivity growth in Bangladesh” is substantive and is within the scope of the journal. In general, the paper is thoroughly researched and well-written. By the way, I have some minor comments for the authors that would help to improve the quality of the manuscript.

Response to comment #1: We would like to express our gratitude for your valuable and insightful suggestions and comments regarding the improvement of our manuscript. We firmly believe that they have significantly enhanced the scientific value of the manuscript, and we sincerely appreciate your contributions.
**Abstract**

C**omment #2:** Line # 35: Authors wrote “… performed a spatial-temporal mapping of the area, production, and yield….”. Is the word “area” sufficient or it is “cultivated/cultivation area”?

Response to comment #2: Thank you for the suggestion. The word “cultivation” has been added.

**Comment #3:** Line # 37 – 38: Replace “Results show that …” with “Results showed that….”.

Response to comment #3: The word 'show' has been replaced with 'showed'.

**Comment #4:** Line # 41: “…the rice area in 19 districts, 11 districts, and 13 districts declined significantly”. The word “rice area” is not a suitable wording. Please replace it with “rice-cultivation-area” or “cultivation area”.
Response to comment #4: The word "cultivation" has been added before the word "area".

**Introduction**

**Comment #5:** Line # 60-63: Please add the information “over 3.5 billion people are solely dependent upon rice for at least 20% of their daily required calories” from Introduction section of “Alam, M. J., Alamin, M., Sultana, M. H., Ahsan, M. A., Hossain, M. R., Islam, S. S., & Mollah, M. N. H. (2020). Bioinformatics studies on structures, functions and diversifications of rolling leaf related genes in rice (Oryza sativa L.). Plant Genetic Resources, 18(5), 382-395” with the sentence “With over half of the world's population depending on rice for their daily energy, and it supplies approximately 62% carbohydrate, 46% protein, 8% fat, 7% calcium, and 44% phosphorus of the recommended dietary allowance” and cite Alam et al. 2020.

Response to comment #5: We appreciate your suggestion, and we have included the provided information and incorporated the reference you suggested. Thank you for your valuable input.
**Comment #6:** Line # 65-66: Please add citation for the sentence “China is the leading rice-producing country, followed by India, Bangladesh, and Vietnam”.

Response to comment #6: We appreciate your observation. The citation for the sentence "China is the leading rice-producing country, followed by India, Bangladesh, and Vietnam" has been duly included.
**Comment #7:** Line #103-104: “This paper analyzed spatiotemporal” is not a standard wording. Please reshape the sentence as “In this study, we analyzed spatiotemporal data on cultivation area, and production and yield of rice to examine/investigate the trends and growth patterns from 2006-2007 to 2019-2020 in Bangladesh. Do not mention the data sources under Introduction section, rather write it under Materials and Methods section.
Response to comment #7: Thank you for the suggestion. We have revised the sentence accordingly.
**Materials and Methods**

**Comment #8:** Line # 120: Replace the word “area” with “cultivation area”. Also, this is applicable for whole body of this manuscript.

Response to comment #8: We have made the necessary adjustment by replacing the term "area" with "cultivation area" throughout the entire manuscript, as suggested.

**Comment #9:** Please add a sentence to mention the level of significance considered for different statistical tests (e.g., normality test, t-test, etc.) under “Statistical analysis” subsection.

Response to comment #9: As per your recommendation, we have included the significance level of 5% in the analysis. Thank you for the suggestion.
**Results**

**Comment #10:** 1. Line # 233: “… seasons in Bangladesh is illustrated in Fig 4”. Replace “is” with “are”.

Response to comment #10: The word 'is' has been replaced with 'are'.

**Comment #11:** 2. Line # 293: Add unit of measurement for area, production and yield within brackets.

Response to comment #11: We have incorporated the percentage as a unit of measurement for the growth rate.

**Comment #12:** 3. Line # 425: Replace “will” with “would” in line # 425.

Response to comment #12: The word 'will' have been replaced with 'would'.

**Discussion**

**Comment #13:** Line # 484: Add an “and” before the phrase “declined over time”.

Response to comment #13: We have corrected it accordingly.

**Comment #14:** Line # 485: Replace “rice area” with “rice cultivation areas”.

Response to comment #14: We have corrected it accordingly.

**Comment #15:** Line # 555: Add “were” verb before the phrase “revealed in five ….” in line no. 555.

Response to comment #15: We have added ‘were’ verb before the phrase “revealed in five ….” accordingly.

**Comment #16:** Line # 561: Replace “will” with “would” in line no. 561.

Response to comment #16: The word 'will' have been replaced with 'would'.
**Comment #17:** I would suggest to add practical implications of this research under the Discussion section.

Response to comment #17: Thank you for the suggestion. We have made an effort to incorporate the practical implications of this research into the discussion section. Please see the text, “*The research findings have significant practical implications for informing and guiding future agricultural practices and policies in rice-intensive areas of Bangladesh. The study provides valuable insights into the dynamic changes in the area and production of rice cultivation at a more disaggregated level, representing diverse ecosystems and management constraints. Policymakers can utilize this information to develop targeted strategies and interventions aimed at enhancing rice production in specific regions. For instance, regions with favorable environmental conditions and fertile soil content can be encouraged to prioritize expanding rice cultivation. Efforts can be directed towards providing farmers in these regions with access to modern high-yielding varieties and advanced agricultural technologies to improve productivity. Conversely, areas facing challenges such as salinity or hilly terrain require tailored approaches to overcome these limitations, such as the development of stress-tolerant rice varieties and the implementation of appropriate irrigation systems. Additionally, identifying regions where rice production is declining, or farmers are shifting to alternative crops can guide initiatives aimed at revitalizing rice cultivation through improved irrigation facilities, training programs, and the development of short-duration varieties. The study highlights the areas that require closer attention to overcome challenges and enhance productivity, ultimately contributing to achieving Sustainable Development Goal 2.3 of doubling agricultural productivity and income for smallholder farmers by 2030. In summary, these practical implications can contribute to sustainable agricultural practices, enhance food security, and improve the livelihoods of farmers in Bangladesh.*”

**Comment #18:** In the Discussion section limitation of this study and future scope could be added.
Response to comment #18: Thank you for the suggestion. We have made an effort to incorporate the limitation and future research scope into the discussion section. Please see the text, “*Despite the valuable insights provided by this study, there are certain limitations that should be acknowledged. First, this research focused primarily on the quantitative analysis of rice cultivation area and production trends, and did not delve into the underlying socio-economic factors that may influence these trends. Additionally, this study relied on secondary data sources, which may be subject to limitations such as data accuracy and availability. Conducting primary data collection through field surveys and interviews could enhance the accuracy and reliability of the findings. Furthermore, the study's scope was limited to a specific timeframe (2006-2019), and it is important to recognize that future changes in climate, technology, and agricultural practices could impact rice cultivation in unforeseen ways.*

*Building on the findings and limitations of this study, there are several areas for future research that can further contribute to the understanding of rice cultivation in Bangladesh. Firstly, investigating the socio-economic factors that influence farmers' decision-making processes regarding rice cultivation, including factors such as market conditions, government policies, and farmers' preferences, would provide valuable insights into the drivers of rice production. Secondly, exploring the impact of climate change on rice cultivation, including the effects of rising temperatures, changing rainfall patterns, and increased occurrences of extreme weather events, is crucial for developing climate-resilient agricultural strategies. Additionally, examining the adoption and effectiveness of specific interventions aimed at improving rice productivity, such as the dissemination of high-yielding varieties, the implementation of irrigation systems, and the provision of training and extension services, would help in identifying best practices and areas for improvement. Finally, integrating remote sensing and geospatial analysis techniques can enhance the accuracy and timeliness of monitoring rice cultivation dynamics, allowing for more precise and up-to-date assessments of cultivation area and production trends.*”

**Conclusion**

**Comment #19:** Line 545-550: First four sentences of Conclusion section are repetition that are already told in objective and method section. Rewrite these into one introductory sentence of Conclusion and write the conclusion with more focus on main finding and recommendation and policy making for government.

Response to comment #19: We greatly appreciate your valuable suggestion, which has prompted us to thoroughly revise the conclusion section and incorporate essential policy recommendations.

Thank you once again for your precious comments and advice. Those comments are all valuable and very helpful for revising and improving our manuscript. We have revised the manuscript accordingly, and our point-by-point responses are presented above. We hope you are satisfied with our answers and the new data we have provided. Our deepest gratitude goes to you for your careful work and thoughtful suggestions that have helped improve this paper substantially.

Sincerely yours

*All Authors’*
