# Supplementary material for: Spatiotemporal mapping of rice acreage and productivity growth in Bangladesh
Source: PLoS One. 2024 Mar 15;19(3):e0300648. doi: 10.1371/journal.pone.0300648 (PMC10942088; doi:10.1371/journal.pone.0300648)
Supplement: Attachment — Submitted filename: Response to Reviewer 4.docx [file pone.0300648.s004.docx]

**Manuscript number:** PONE-D-23-09867R1

**Title:** Spatiotemporal mapping of rice acreage and productivity growth in Bangladesh

**Journal:** PLOS ONE

Dear Reviewer,

Thank you for the comments concerning our manuscript. We deeply appreciate your positive evaluation of our work. Those comments are valuable and very helpful. We have read through comments carefully and have made corrections. Please see below; all tasks and re-visions taken are shown point-by-point.

**Response to Reviewer’s comments**

**Reviewer #4:**

**Comment #1:** If the authors have adequately addressed your comments raised in a previous round of review and you feel that this manuscript is now acceptable for publication, you may indicate that here to bypass the “Comments to the Author” section, enter your conflict of interest statement in the “Confidential to Editor” section, and submit your "Accept" recommendation.

**Reviewer response:** All comments have been addressed

Response to comment #1: Thank you for your comment. We appreciate your positive feedback.

**Comment #2:** Is the manuscript technically sound, and do the data support the conclusions?

The manuscript must describe a technically sound piece of scientific research with data that supports the conclusions. Experiments must have been conducted rigorously, with appropriate controls, replication, and sample sizes. The conclusions must be drawn appropriately based on the data presented.

**Reviewer response:** No

Response to comment #2: Thank you for your comment. Indeed, our primary emphasis was on delving into the regional context, striving to extract meaningful insights within the framework of 64 specific locations. It's crucial to acknowledge that the rice-growing ecosystem in Bangladesh exhibits considerable diversity, influenced by factors such as geographical position, socio-economic conditions, and environmental variations. Consequently, the previous study's findings have limitations in terms of statistical robustness and generalizability for shaping region-specific policies. In response to these limitations, our current study adopts a more granular approach by analyzing data from 64 locations. This approach aims to offer a comprehensive understanding of the dynamics of rice cultivation in Bangladesh. We believe that this shift not only enhances the statistical validity of our research but also facilitates the formulation of more effective policies tailored to specific locations. For the technical soundness and scientific evidence, please refer to results and discussion sections for details.

In addressing the data support aspect, we employed information related to rice cultivation area, production, and yield over a 14-year period across 64 districts, covering the Aus, Aman, and Boro seasons from 2006-2007 to 2019-2020. The data were sourced from the Bangladesh Bureau of Statistics (BBS), a government department with national authorization, which consistently collects rice area, production, and yield data for all 64 districts annually since 2006. Consequently, our study's sample initiation was from 2006, extending up to the latest available data, ensuring the utilization of a nationally representative data source in Bangladesh. Regarding sample size, our time series dataset comprises 2688 observations or data points for each variable, establishing a comprehensive and nationally representative sample.

In the context of replicating our study, our methodology can be applied to datasets in other rice-growing countries and diverse crops. Despite revealing potential threats to agricultural sustainability, such as temporal variations and regional disparities in rice production, these aspects have received limited exploration. Consequently, other nations cultivating rice can replicate our methodology to investigate the dynamics of regional rice cultivation in their respective contexts.

Our study's conclusions, succinctly presented in the conclusion and abstract sections, highlight significant findings from the analyzed data. Notably, we like to refers the following text for your reference, “*To ensure the sustainability of rice production in Bangladesh, it is imperative to have a comprehensive understanding of the spatiotemporal distribution of rice cultivation area, production, and yield. This knowledge will enable the formulation of region-specific policies tailored to the specific needs of different areas. The study has introduced novel methodological approaches for trend analysis and spatial clustering. Our findings showed that 14 years averages of rice cultivation area, production, and yield for three major seasons, Aus, Aman, and Boro, differ significantly among the study districts in Bangladesh. The Aus season has the highest temporal variability of rice production determinants, followed by the Aman and Boro seasons. Regional disparities in production were revealed in five cluster groups for the Aus season, seven for the Aman, and six for the Boro season. The share of HYV adoption significantly increased for most of the season. A significant increasing trend in Aus (0.007-0.521%), Aman (0.004-0.039%), and Boro (0.013-0.584%) were observed in 28, 34, and 36 districts, respectively, with an increase of 1% adoption of HYV. Predictions revealed that more than 5% of rice production would be increased in 28 districts in the Aus season, and for Aman and Boro, more than 5% would be increased in Narail and Bogura, respectively. Moreover, a 1-5% increase will be found in 50, 54, and 41 districts in Aus, Aman, and Boro seasons, respectively. These findings underscore the importance of formulating tailored and targeted policies at the regional level to effectively enhance rice productivity in Bangladesh*”.

We sincerely appreciate your insightful comments, and in light of your input, we have taken steps to incorporate the novelty of our research into the introduction section of our manuscript. We hope you are satisfied with our response, and if necessary, we are happy to provide further clarification.

**Comment #3:** Has the statistical analysis been performed appropriately and rigorously?

**Reviewer response:** No

Response to comment #3: We applied a robust and rigorous statistical method for data analysis. Augmented Dickey-Fuller test and Shapiro-Wilk normality test were applied to check the stationarity of time series and normality test of the respective data series. In growth analysis, we employed exponential growth model to estimate growth and trend nature. To achieve more comprehensive and precise results, we utilized time series data of rice cultivation area, production, and yield at the district level as input parameters for clustering. We employed robust multivariate clustering techniques, specifically dynamic time warping (DTW). This approach had overcome the limitations in identifying regional heterogeneity as it focused on mean values and had a limited scope. Additionally, we employed principal component analysis and optimal clustering methods to identify the most suitable clusters in our study. The cluster analysis resulted in grouping similar districts, facilitating the creation of a rice zoning map and providing valuable insights for policy implications. Moreover, we employed Ordinary Least Squares (OLS) regression to investigate the adoption rate and rice production in Bangladesh, aiming to evaluate the influence of adopting high-yielding varieties on rice production. Please see the details in the “Materials and Methods” section.

**Comment #4:** Have the authors made all data underlying the findings in their manuscript fully available?

The PLOS Data policy requires authors to make all data underlying the findings described in their manuscript fully available without restriction, with rare exception (please refer to the Data Availability Statement in the manuscript PDF file). The data should be provided as part of the manuscript or its supporting information, or deposited to a public repository. For example, in addition to summary statistics, the data points behind means, medians and variance measures should be available. If there are restrictions on publicly sharing data—e.g. participant privacy or use of data from a third party—those must be specified.

**Reviewer response:** No

Response to comment #4: We have uploaded the minimal anonymized dataset and subsequently adjusted the data availability statement according to the journal data availability guideline. Please see the PLOS ONE data availability guideline.

**Comment #5:** Is the manuscript presented in an intelligible fashion and written in standard English?

PLOS ONE does not copyedit accepted manuscripts, so the language in submitted articles must be clear, correct, and unambiguous. Any typographical or grammatical errors should be corrected at revision, so please note any specific errors here.

**Reviewer response:** No

Response to comment #5: We acknowledge the reviewer's valuable feedback regarding the need for a thorough check for grammatical and typographical errors throughout the manuscript. We have carefully addressed this concern and made the necessary revisions to ensure the quality and accuracy of the text. Thank you for your thoughtful input.

**Comment #6:** Review Comments to the Author

Please use the space provided to explain your answers to the questions above. You may also include additional comments for the author, including concerns about dual publication, research ethics, or publication ethics. (Please upload your review as an attachment if it exceeds 20,000 characters)

**Reviewer response:** Sorry to say that there is nothing new in the manuscript. A lot of similar studies have been performed. These cited studies are missing in this paper. I suggest rejecting this paper due to lack of novelty.

Response to comment #6: Thank you for your comment. While we appreciate your perspective, we believe there are unique contributions in our manuscript that may not have been fully captured in the previous studies. We acknowledge the importance of novelty in research and have carefully revised our manuscript to emphasize the distinctive aspects of our work in the introduction section. We have also incorporated relevant citations to address the updated references. We hope that upon reevaluation, you may find the revised manuscript to be a valuable addition to the existing literature. For detailed insights into the novelty of our research, kindly refer to response of Comment #1 from Reviewer #1 and Introduction section of our manuscript. Your insights have been instrumental in shaping our improvements, and we appreciate your time and consideration.

Sincerely yours

*All Authors’*
