# Supplementary material for: Spatiotemporal mapping of rice acreage and productivity growth in Bangladesh
Source: PLoS One. 2024 Mar 15;19(3):e0300648. doi: 10.1371/journal.pone.0300648 (PMC10942088; doi:10.1371/journal.pone.0300648)
Supplement: Attachment — Submitted filename: Reviewer Comments.docx [file pone.0300648.s002.docx]

The research topic “Spatiotemporal mapping of rice acreage and productivity growth in Bangladesh” is substantive and is within the scope of the journal. In general, the paper is thoroughly researched and well-written. By the way, I have some minor comments for the authors that would help to improve the quality of the manuscript.

**Abstract:**

1. Line # 35: Authors wrote “… performed a spatial-temporal mapping of the *area*, production, and yield….”. Is the word “area” sufficient or it is “cultivated/cultivation area”?
2. Line # 37 – 38: Replace “Results **show** that …” with “Results **showed** that….”.
3. Line # 41: “…the *rice area* in 19 districts, 11 districts, and 13 districts declined significantly”. The word “rice area” is not a suitable wording. Please replace it with “rice-cultivation-area” or “cultivation area”.

**Introduction:**

1. Line # 60-63: Please add the information *“over 3.5 billion people are solely dependent upon rice for at least 20% of their daily required calories”* from Introduction section of *“Alam, M. J., Alamin, M., Sultana, M. H., Ahsan, M. A., Hossain, M. R., Islam, S. S., & Mollah, M. N. H. (2020). Bioinformatics studies on structures, functions and diversifications of rolling leaf related genes in rice (Oryza sativa L.). Plant Genetic Resources, 18(5), 382-395”* with the sentence *“With over half of the world's population depending on rice for their daily energy, and it supplies approximately 62% carbohydrate, 46% protein, 8% fat, 7% calcium, and 44% phosphorus of the recommended dietary allowance”* and cite Alam et al. 2020.
2. Line # 65-66: Please add citation for the sentence “China is the leading rice-producing country, followed by India, Bangladesh, and Vietnam”.
3. Line #103-104: “This paper analyzed spatiotemporal” is not a standard wording. Please reshape the sentence as “In this study, we analyzed spatiotemporal data on cultivation area, and production and yield of rice to examine/investigate the trends and growth patterns from 2006-2007 to 2019-2020 in Bangladesh. Do not mention the data sources under Introduction section, rather write it under Materials and Methods section.

**Materials and Methods:**

1. Line # 120: Replace the word “area” with “cultivation area”. Also, this is applicable for whole body of this manuscript.
2. Please add a sentence to mention the level of significance considered for different statistical tests (e.g., normality test, t-test, etc.) under “Statistical analysis” subsection.

**Results:**

1. Line # 233: “… seasons in Bangladesh is illustrated in Fig 4”. Replace “is” with “are”.
2. Line # 293: Add unit of measurement for area, production and yield within brackets.
3. Line # 425: Replace “will” with “would” in line # 425.

**Discussion:**

1. Line # 484: Add an “and” before the phrase “declined over time”.
2. Line # 485: Replace “rice area” with “rice cultivation areas”.
3. Line # 555: Add “were” verb before the phrase “revealed in five ….” in line no. 555.
4. Line # 561: Replace “will” with “would” in line no. 561.
5. I would suggest to add practical implications of this research under the Discussion section.
6. In the Discussion section limitation of this study and future scope could be added.

**Conclusion:**

1. Line 545-550: First four sentences of Conclusion section are repetition that are already told in objective and method section. Rewrite these into one introductory sentence of Conclusion and write the conclusion with more focus on main finding and recommendation and policy making for government.
